# Diagnostic accuracy of three tests for *Salmonella Typhi* infection in adult patients presenting to health facilities in an informal urban settlement in Nairobi, Kenya

**DOI:** 10.1016/j.pmedr.2025.103278

**Published:** 2025-10-15

**Authors:** Anthony K. Nganga, Marshal M. Mweu

**Affiliations:** Department of Public and Global Health, Faculty of Health Sciences, University of Nairobi, Nairobi, Kenya

**Keywords:** Diagnostic accuracy, *Salmonella Typhi*, Widal test, Salmonella antigen test, Rapid antibody test

## Abstract

**Objective:**

Accurate and timely diagnosis of typhoid fever is undermined by the absence of reliable reference test(s). The objective here was to estimate the sensitivity (Se), specificity (Sp), negative and positive predictive values (NPV and PPV) of the Widal, *Salmonella Typhi* (*S. typhi*) rapid antigen (Accurate^R^ Salmonella Antigen Test kit (SAT)) and antibody (OnSite Typhoid IgM/IgG Combo Rapid Test (Rapid Ab)) tests for the diagnosis of acute *S. typhi* infection in Kenya.

**Methods:**

This cross-sectional study enrolled patients symptomatic of typhoid fever at three health facilities situated within an informal urban settlement in Nairobi County, Kenya. Using the patients' diagnostic data, a Bayesian latent class model was used to infer the tests' diagnostic accuracy.

**Results:**

The Rapid Ab test recorded the highest Se (94.9% [95% PCI 76.1, 99.8]) compared to Widal (59.5% [95% PCI 48.6, 77.9]) and SAT (54.2% [95% PCI 43.5, 64.8]). The assay's Sp (95.9% [95% PCI 85.0, 99.8]) was statistically similar to Widal (85.9% [95% PCI 80.8, 91.7] but superior to SAT (67.1% [95% PCI 60.7, 73.0]). PPVs for the Rapid Ab and Widal tests were comparable but distinct from SAT. NPV for the Rapid Ab test was the highest.

**Conclusion:**

The Rapid Ab assay lends itself as a suitable standalone diagnostic to guide surveillance, diagnosis and treatment of acute typhoid fever in high-burden, resource-limited settings.

## Highlights

• The Rapid antibody test exhibits high sensitivity and specificity for *Salmonella Typhi*.
• The Rapid antibody assay posts strong negative and positive predictive values.
• The assay lends itself to diagnosis and treatment of acute typhoid fever in poor settings.

## 1 Introduction

Typhoid fever is a systemic gastro-intestinal infection caused by *Salmonella enterica* serotype Typhi *(S. typhi*) bacteria (Andualem et al., 2014). Transmission is primarily through the faecal-oral route following ingestion of contaminated beverage and/or food. The infection is rarely fatal but if untreated, potentially life-threatening complications can arise (Andualem et al., 2014; Bhandari et al., 2025; Radhakrishnan et al., 2018), with case fatality rate exceeding 10% (Akullian et al., 2015).

The symptomatology of an *S. typhi* infection is non-specific and indistinguishable from other febrile illnesses (Bhandari et al., 2025). Consequently, reaching a definitive diagnosis without a confirmatory test is impracticable. Aetiological diagnosis of infected individuals in an accurate and timely manner is critical, regardless of a subject's symptomatic status. Importantly, reliable diagnostic tests should effectively discriminate between infected and uninfected individuals so as to not only inform prompt therapy and thereby minimise potential spread of *S. typhi* infection (Ajibola et al., 2018), but also avert unnecessary antibiotic prescriptions.

Conventionally, presumptive diagnosis of typhoid fever has been made using the Widal test (Bakr et al., 2011) – a serological test that relies on the demonstration of rising antibody titres following *S. typhi* infection. The test is fairly affordable and has considerably minimal laboratory requirements, explaining why it still finds application in resource-limited settings. However, it is plagued by low sensitivity (Se) and specificity (Sp) that vary geographically (Andualem et al., 2014; Bhutta, 2006; Ley et al., 2010; Olopoenia and King, 2000; Parry et al., 2011). Moreover, the absence of a universal cut-off defining a positive Widal test allows for varied interpretation of results (Ajibola et al., 2018); an important element considering that the Se for the test is dependent on the cut-off values used (Omuse et al., 2010). The test is also time consuming and there is notable brand variation in Se and Sp of the antigen used (Bakr et al., 2011).

In resource-limited endemic settings, the utility of rapid diagnostic tests as point of care diagnostics is growing (Sapkota et al., 2023). In Kenya, the available rapid diagnostic tests detect either the *S. typhi* antigen (SAT) or *S. typhi* antibody (Rapid Ab test). SAT is a rapid, one-step immunochromatographic assay that selectively identifies *S. typhi* infection by utilizing antibodies specific to components of the outer membrane of *S. typhi* such as the lipopolysaccharide, O or H antigens (Wijedoru et al., 2017). The test can qualitatively detect *S. typhi* antigen in human stool, serum or plasma. The Rapid Ab test is equally a lateral flow assay that detects IgM and IgG antibodies to *S. typhi* in serum, plasma or whole blood (Kawano et al., 2007; Wijedoru et al., 2017). Owing to their simplicity of usage and rapidity, rapid diagnostic tests are suitable in settings where equipment and/or trained personnel are in short supply (Parry et al., 2011). Nevertheless, rapid diagnostic tests suffer variations in Se and Sp – with potential cross-reactivity with other *Salmonella* antigens (Kuijpers et al., 2018) undermining the tests' specificities.

A key shortcoming in the evaluation typhoid fever diagnostics stems from the absence of a perfect reference standard capable of classifying infected and uninfected individuals without error (Arora et al., 2019). Evaluation of index tests using imperfect references may bias the tests' accuracy estimates (Enøe et al., 2000). In the absence of an appropriate reference test(s), Bayesian latent class models can be employed to simultaneously derive the Se and Sp of two or more tests without knowledge of the true infection status (Johnson et al., 2019; Joseph et al., 1995). Thus, the objective of this study was to estimate the Se and Sp as well as the predictive values (negative (NPV) and positive (PPV)) of Widal, SAT and Rapid Ab tests for the diagnosis of acute *S. typhi* infection in Kenya.

## 2 Methods

### 2.1 Study setting, design and population

The study was conducted at three state-run health facilities located within the Kibra informal settlement in Nairobi County, Kenya: Kibra Community Health Centre - Amref, Kibra DO Dispensary and Langata Health Centre. The choice of these facilities was premised on their broad range of outpatient health services (including typhoid fever diagnostics) that they render to a large, predominantly low-socioeconomic, urban population burdened by typhoid fever. The facilities' monthly patient load averages ∼1200 patients.

A facility-based cross sectional study design was utilized to derive the accuracy of the tests for *S. typhi* infection. This design was rationalised given the study's descriptive nature.

### 2.2 Sample size and participant selection

The McNemar's formula for paired proportions was employed to estimate the required sample size (Connor, 1987; Fosgate, 2009):

$$n={\left(\frac{Z_{\alpha /2}\sqrt{p_{\text{disc}}}+Z_{\beta }\sqrt{p_{\text{disc}}-p_{\text{diff}}^{2}}}{p_{\text{diff}}}\right)}^{2}$$

$$p_{\text{disc}}=\left(1-{\mathrm{Se}}_{1}\right)+\left(1-{\mathrm{Se}}_{2}\right)$$

$$p_{\text{diff}}=\left(1-{\mathrm{Se}}_{1}\right)-\left(1-{\mathrm{Se}}_{2}\right)$$

where: n= sample size required for each test, $Z_{\frac{\alpha }{2}}$ (1.96) is the critical value required for the two-sided 95% confidence level, and $Z_{\beta }$ (0.84) is the critical value specifying the desired power of 80%. ${\mathrm{Se}}_{1}$ and ${\mathrm{Se}}_{2}$ are Se estimates of Widal and Rapid Ab test for *S. typhi* respectively, obtained from literature: Se_1_ set at 0.61 (Aquino et al., 1991) and Se_2_ set at 0.71 (Kawano et al., 2007). Given these figures, a total sample of 394 subjects was targeted for enrolment into the study following an upward adjustment by 5% to account for non-response.

To obtain the requisite sample, eligible patients presenting to the three facilities between October 2023 and April 2024 were systematically randomly sampled based on their time of arrival at the specific facility. Specifically, following an initial random start, subsequent patients were identified by adding a pre-determined sampling interval (obtained by dividing an expected day's total patient turnout by the number to be sampled on that day) until the required sample for a specific day was attained. Of note, eligibility was limited to patients aged ≥14 years who satisfied the clinical criteria for suspected typhoid fever: febrile (history of fever >3 days and demonstrable pyrexia ≥37.5 °C) and presenting with at least one of the following: headache, malaise, myalgia, abdominal symptoms (discomfort, diarrhoea or constipation) and, at the time of the study, resided in an identifiable location within Kibra. Recruitment commenced immediately following triage by the nurse and examination by the resident clinician. The potential participants were subsequently briefed about the study prior to giving consent.

### 2.3 Ethical considerations

Written permission to conduct the study was secured from the Kenyatta National Hospital - University of Nairobi Ethics and Research Committee (Ref. No. KNH/ERC/A/159), the Nairobi City County (Ref. No. NCCG/HWN/REC/432) and the National Commission for Science, Technology and Innovation (Ref. No. NACOSTI/P/23/28921). Additionally, written informed consent was secured from individual patients prior to enlisting them in the study.

### 2.4 Sample collection

Upon recruitment, trained laboratorians from the participating facilities obtained blood and stool samples from each enrolled subject after which, they were processed and ran through the three tests as per standard laboratory procedures for *S. typhi* diagnosis. Besides the samples, the participants' sociodemographic characteristics (age, sex, employment and marital statuses, education level, sources of water and accessibility to toilet facilities) were captured via an orally-administered semi-structured questionnaire.

### 2.5 Target condition

The infection (latent) status targeted by the three tests represents a stool and/or blood sample containing either the *S. typhi* antigen or its antibodies at any concentration.

### 2.6 Widal test

The test was performed as per standard guidelines for quantitative tube Widal test (Cat No. 8.01.25.2.0010, Atlas Medical GmbH, Blankenfelde-Mahlow, Germany). Briefly, a 2 ml mixture of freshly prepared serum and normal saline was dispensed into two series of seven test tubes each specific to the O and H antigens of *S. typhi*. Moreover, a 1 ml saline solution in a separate tube served as a control for each of the series. Subsequently, single drops of *Salmonella* O and H antigen suspensions were added to the separate series accordingly. On gentle agitation, the tubes were covered and incubated overnight at 37 °C. The highest dilution displaying a compact, granular agglutination for the O antigen series and a floccular appearance for the H antigen series was noted. As per the manufacturer's manual, a titre ≥1:80 for the “O” antigen and ≥1:160 for the “H” antigen was deemed positive for *S. typhi* infection.

### 2.7 SAT

The Accurate^R^ Salmonella Antigen Test kit (Lot no. 8200P, Huichao Medicals, China) was used for this assay (primarily detects the bacterium's lipopolysaccharide antigen), with testing performed as guided by the manufacturer. Briefly, 50 g of a stool specimen was added to the dilution tube containing extraction buffer and mixed vigorously. The cassette was removed from the pouch and labelled. Subsequently, three drops of the mixture were added into the sample well. Results were read within 15 min - a positive test was denoted by the appearance of two coloured bands in the membrane.

### 2.8 Rapid Ab test

The OnSite Typhoid IgM/IgG Combo Rapid Test (Ref. R0160C, CTK Biotech, USA) was employed to screen for the presence of IgM and/or IgG antibodies in collected blood samples. The assay, which utilises the O and H antigens from *S. typhi* to maximize sensitivity, was carried out as per manufacturer instructions. The cassette was removed from the pouch and labelled. A drop of whole blood from a fingertip puncture was dispensed into the sample well and mixed with the sample diluent. Results were read after 15 min, with a positive test result indicated by the appearance of a coloured band on the control region plus either of the following: (a) a coloured band on the M region (IgM reactive) or (b) a coloured band on the G region (IgG reactive) or (c) the coloured bands on both M and G regions (IgM and IgG reactive).

### 2.9 Statistical analysis

Initially, the sociodemographic characteristics of the respondents were summarised using medians and ranges for continuous variables and frequencies and percentages for categorical variables.

A Bayesian latent class model framework was utilized to infer the Se, Sp and predictive values of the three tests under evaluation. Notably, the reporting design adopted in this study conformed to published standards for reporting of diagnostic accuracy studies that use Bayesian latent class models (Kostoulas et al., 2017).

To fit a Bayesian latent class model, three assumptions are needful (Hui and Walter, 1980). Firstly, the target population should consist of at least two subpopulations with differing prevalences. However, in situations where only a single population exists, as in the current study, at least three tests are necessary to achieve model identifiability (Toft et al., 2005). Secondly, the Se and Sp of the tests under evaluation should be constant across the subpopulations. Granted this study's single population, the constancy assertion was arguably upheld. Thirdly, the tests should be conditionally independent given the infection status. We considered this assumption satisfied given that the three tests bear different targets: antibodies to O (lipopolysaccharide) and H (flagellin) antigens (Widal test), specific *Salmonella* antigens (SAT) and IgM and/or IgG antibodies (Rapid Ab). Statistically, this implies that the probability of testing either positive or negative to any of the assays should be independent of the outcome of the other tests.

Counts (O), of the different test combinations (e.g. +,+,+) were presumed to be multinomially distributed:

$$O|{\mathrm{Se}}_{k}{\mathrm{Sp}}_{k}P∼\text{multinomial}\left(\text{prob},n\right)$$

where ${\mathrm{Se}}_{k}$ and ${\mathrm{Sp}}_{k}$ represent the characteristics for test $k\;\left(k=1:3\right)$ and P is the true prevalence of *S. typhi* for the study population. Prob is a vector of probabilities of observing the different combinations of test results and n is the total sample size. For instance, for a subject testing positive to each of the three tests, prob is indicated by:

$$\mathrm{prob}=\mathrm{Pr}\left(T_{1}^{+}T_{2}^{+}T_{3}^{+}|D^{+}\right)+\mathrm{Pr}\left(T_{1}^{+}T_{2}^{+}T_{3}^{+}|D^{-}\right)={\mathrm{Se}}_{1}{\mathrm{Se}}_{2}{\mathrm{Se}}_{3}P+\left[1-{\mathrm{Sp}}_{1}\right]\left[1-{\mathrm{Sp}}_{2}\right]\left[1-{\mathrm{Sp}}_{3}\right]\left[1-P\right]$$

The single population furnished a total of seven degrees of freedom sufficient to estimate seven parameters (P as well as Se, Sp for each of the tests). Uninformative priors ($\mathrm{beta}\left(11\right)$) were specified for the model parameters since no reliable prior information was available for any of the tests.

To quantify the overall diagnostic performance of the tests, a Youden's index (${\mathrm{Youden}}_{k}$) was computed for each test (Ruopp et al., 2008); the test yielding the highest score being deemed the most accurate:

$${\text{Youden}}_{k}=\left({\mathrm{Se}}_{k}+{\mathrm{Sp}}_{k}\right)-1$$

PPV and NPV specific for test k were computed as follows:

$${\mathrm{PPV}}_{k}={\mathrm{PSe}}_{k}/\left({\mathrm{PSe}}_{k}+\left[1-P\right]\left[1-{\mathrm{Sp}}_{k}\right]\right)$$

$${\mathrm{NPV}}_{k}=\left[1-P\right][{\mathrm{Sp}}_{k}/\left(P\left[1-{\mathrm{Se}}_{k}\right]+\left[1-P\right]{\mathrm{Sp}}_{k}\right)$$

Two Markov Chain Monte Carlo chains with different starting values were used to initiate the model. Each chain contained 70,000 samples – the first 20,000 being discarded as the burn-in. Convergence of the chains was assessed by visualization of trace plots of selected variables and the Gelman-Rubin diagnostic plots. The goodness-of-fit of the Bayesian model was evaluated using the posterior predictive *p*-value. The value is estimated from the difference between the deviance of the observed dataset and that of predicted data generated randomly from the fitted model; values in the region of 0.50 suggestive of an overall good fit (Gelman et al., 2013). The posterior distributions of the test estimates (Se, Sp and predictive values) were reported using the median and the corresponding 95% posterior credible intervals (PCI). Statistical differences between the tests' accuracy estimates were evaluated using a Bayesian *p*-value. The Bayesian model was fitted in OpenBUGS software v3.2.2 (Lunn et al., 2009), but called from R software via the ‘BRugs’ package (Thomas et al., 2006).

## 3 Results

Out of the estimated 394 individuals, 95.4% (n=376) were enrolled into the study. A total of 18 subjects were excluded for either not being able to provide the two required samples or declining consent.

The sociodemographic characteristics of the study participants are captured in Table 1. The median age of the participants was 24 years (Range: 14–55). A majority of the participants (54.5%, n=205) had a secondary level education. About a quarter of the respondents (26.1%, n=98) had access to piped water. Notably, only 19.1% (n=72) of them had access to toilet facilities.

**Table 1:** Summary statistics of the sociodemographic characteristics of adult participants presenting to health facilities in Kibra, Nairobi County, Kenya (n=376).

Variable	Values	Median	Range	Frequency (%)
Age (in years)	–	24	14–55	–
Sex	Male	–	–	182 (48.4)
	Female	–	–	194 (51.6)
Education status	No formal education	–	–	6 (1.6)
	Primary	–	–	110 (29.3)
	Secondary	–	–	205 (54.5)
	Tertiary	–	–	55 (14.6)
Marital status	Single	–	–	149 (39.6)
	Married	–	–	183 (48.7)
	Separated	–	–	37 (9.8)
	Widowed	–	–	7 (1.9)
Source of water	Ground	–	–	134 (35.6)
	Piped	–	–	98 (26.1)
	Surface	–	–	1 (0.3)
	Rain	–	–	143 (38.0)
Employment status	Employed	–	–	138 (36.7)
	Unemployed	–	–	238 (63.3)
Accessibility to toilet	Accessible	–	–	304 (80.9)
	Inaccessible	–	–	72 (19.1)

The cross-classified outcomes of the three tests for the diagnosis of *S. typhi* are displayed in Table 2. In particular, 8.8% (n=33) of the participants registered positive results to all the tests.

**Table 2:** Cross-tabulated results for Widal, *Salmonella Typhi* (*S. typhi*) rapid antigen (SAT) and antibody (Rapid Ab) tests for diagnosis of *S. typhi* infection among adult patients presenting to health facilities in Kibra, Nairobi County, Kenya (n=376).

population	Test outcomes combinations (Widal; SAT; Rapid Ab)								Total (%)
	+;+;+	+;+;-	+;-;+	-;+;+	+;-;-	-;+;-	-;-;+	-;-;-	
aPop (%)	33 (8.8)	4 (1.1)	34 (9.0)	32 (8.5)	34 (9.0)	79 (21.0)	21 (5.6)	139 (37.0)	376 (100)

a Study population.

Estimates of Se, Sp and predictive values of the three tests for *S. typhi* infection are shown in Table 3. Of the three tests, the Rapid Ab test registered the highest Se (94.9%; 95% PCI [76.1, 99.8]). As for Sp, the Rapid Ab test recorded a higher Sp (95.9%; 95% PCI [85.0, 99.8]) than SAT (67.1%; 95% PCI [60.7, 73.0]) but statistically similar to Widal test (85.9%; 95% PCI [80.8, 91.7]). Overall, the Rapid Ab assay registered the best combination of Se and Sp (89.5%; 95% PCI [69.0, 98.2]).

**Table 3:** Estimates of sensitivity, specificity and predictive values of Widal, *Salmonella Typhi* rapid antigen (SAT) and antibody (Rapid Ab) tests for *Salmonella Typhi* infection among adult patients presenting to health facilities in Kibra, Nairobi County, Kenya.

Parameter	Estimate (95% aPCI)
b${\mathrm{Se}}_{\mathrm{Widal}}$	59.5 (48.6, 77.9)
${\mathrm{Se}}_{\mathrm{SAT}}$	54.2 (43.5, 64.8)
${\mathrm{Se}}_{\mathrm{Rapid}\;\mathrm{Ab}}$	94.9 (76.1, 99.8)
c${\mathrm{Sp}}_{\mathrm{Widal}}$	85.9 (80.8, 91.7)
${\mathrm{Sp}}_{\mathrm{SAT}}$	67.1 (60.7, 73.0)
${\mathrm{Sp}}_{\mathrm{Rapid}\;\mathrm{Ab}}$	95.9 (85.0, 99.8)
dP	30.7 (21.3, 39.8)
e${\mathrm{NPV}}_{\mathrm{Widal}}$	82.6 (74.3, 93.1)
${\mathrm{NPV}}_{\mathrm{SAT}}$	76.9 (66.4, 85.6)
${\mathrm{NPV}}_{\mathrm{Rapid}\;\mathrm{Ab}}$	97.8 (87.0, 99.9)
f${\mathrm{PPV}}_{\mathrm{Widal}}$	64.9 (52.5, 80.6)
${\mathrm{PPV}}_{\mathrm{SAT}}$	42.3 (28.7, 53.4)
${\mathrm{PPV}}_{\mathrm{Rapid}\;\mathrm{Ab}}$	91.1 (64.0, 99.7)
g${\mathrm{Youden}}_{\mathrm{Widal}}$	45.6 (33.3, 64.8)
${\mathrm{Youden}}_{\mathrm{SAT}}$	21.4 (7.9, 33.6)
${\mathrm{Youden}}_{\mathrm{Rapid}\;\mathrm{Ab}}$	89.5 (69.0, 98.2)

a Posterior credible interval.

b Sensitivity.

c Specificity.

d Prevalence.

e Negative predictive value.

f Positive predictive value.

g Youden's index.

Regarding predictive values, Rapid Ab test's PPV was higher (91.1%; 95% PCI [64.0, 99.7]) than that of SAT (42.3%; 95% PCI [28.6, 53.4]) but statistically similar to Widal (64.9%; 95% PCI [52.5, 80.6]). However, as for NPV, the Rapid Ab test yielded the highest estimate (97.8%; 95% PCI [87.0, 99.9]). The true prevalence of *S. typhi* infection in this study setting was 30.7% (95% PCI [21.3, 39.8]). A posterior predictive p-value = 0.428 suggested a good model fit to the data.

## 4 Discussion

Using a Bayesian paradigm, we have inferred the Se, Sp and predictive values of three tests for diagnosis of *S. typhi* infection in an endemic poor-resource setting. The Rapid Ab test displayed a strong and superior Se to both Widal and SAT, but the latter two tests exhibited comparable sensitivities – findings that are corroborated elsewhere. For instance, an Egyptian study comparing the Rapid Ab to the Widal test demonstrated superiority of the Rapid Ab's Se (Aboud et al., 2023). A related study comparing the three diagnostics showcased SAT's better capability at detecting *S. typhi* infection in the initial five days whilst Widal and Rapid Ab tests, by virtue of being immunological tests, exhibited higher Se five days post-infection (Saini and Duggal, 2022). Nonetheless, some studies have reported higher sensitivities of the Widal test when compared to rapid antibody kits (Ousenu and Ali, 2021; Shahapur et al., 2021).

As for Sp, the Rapid Ab and Widal tests displayed strong and similar estimates that were significantly higher than SAT's Sp. Our demonstration of the Rapid Ab test's high Sp is supported by other studies (Aboud et al., 2023; Jahan et al., 2021; Tarupiwa et al., 2015). One of the studies comparing this Rapid Ab kit to Widal test found a Sp estimate of 88.7% (Jahan et al., 2021). In yet another study carried out in Egypt, Sp for the Rapid Ab test was rated at 98.4% (Aboud et al., 2023), further corroborating the findings from our study. In another study, the recorded Sp for the Rapid Ab test was observably high at 94.34% in an evaluation comparing it to another rapid diagnostic test (Tarupiwa et al., 2015).

Overall, the Rapid Ab test gave the best combination of Se and Sp as evidenced by its Youden index (89.5% [95% PCI 69.0; 98.2]). This finding is consistent with reports from other studies which revealed that Rapid Ab tests based on the detection of IgM and IgG antibodies against *S. typhi* yielded a good mix of Se and Sp (Sattar et al., 2014; Tarupiwa et al., 2015). Reliable point-of-care tests should not only have rapid turn-around times but also afford high Se and Sp (>90%), thus enabling prompt and effective clinical decisions. As such, granted its solid performance, the Rapid Ab test affords commendable promise to guide surveillance and management of typhoid fever under field-based conditions in low-resource settings.

False positives and negatives compromise the Sp and Se of diagnostic tests. For SAT, false negatives can arise either due to improper processing of samples or when the test is run at a later phase of an *S. typhi* infection thereby undermining its Se (Saini and Duggal, 2022). As for antibody-based tests, false negatives arising as a result of their use in the earlier phases of an infection can impair the tests' Se (Andualem et al., 2014; Mawazo et al., 2019). Keeping with these tests, false positives attributable to past infections and/or vaccinations as well as potential cross-reactivity with conditions such as malaria can diminish their Sp (Bakr et al., 2011).

Of the three tests, the Rapid Ab registered the highest NPV. Nevertheless, its PPV was higher than SAT but consistent with that of Widal. The Rapid Ab's high predictive values signify strong confidence in both negative and positive results further justifying its suitability in acutely-infected, endemic settings as a standalone diagnostic. This study's predictive value findings for the Rapid Ab and Widal tests closely mirror those observed in a related Egyptian study (Aboud et al., 2023).

A few limitations are inherent in the present study. Firstly, study eligibility relied on a subject's recall of recent vaccination and treatment histories. It is conceivable that some individuals already on treatment for typhoid fever or recently vaccinated against *S. typhi* enlisted in the study. Such false positives would likely reduce the Sp of the tests. Secondly, enrolment of only symptomatic individuals – likely to harbour high bacterial loads and thus post stronger immunological responses – would positively impact the tests' sensitivities. Notwithstanding, since Bayesian latent class models permit the quantification of tests' accuracy devoid of classification errors (Enøe et al., 2000), the findings from this study can be considered readily generalizable to populations with similar burden of *S. typhi*.

## 5 Conclusion

We have derived the Se, Sp and predictive values of Widal, Rapid Ab and SAT for the diagnosis of *S. typhi* infection in a symptomatic outpatient population. Of the tests, the Rapid Ab assay displayed the highest Se. Its Sp was similar to that of Widal but significantly higher than SAT. Correspondingly, Rapid Ab's NPV was the highest, but recorded a comparable PPV with the Widal test. Consequently, the Rapid Ab test renders good promise towards informing surveillance, diagnosis and treatment of acute typhoid fever in endemic settings.

## Data Availability

Data will be made available on request.
